# Effects of exercise interventions in Alzheimer's disease: A meta‐analysis

**DOI:** 10.1002/brb3.3051

**Published:** 2023-06-18

**Authors:** Sagor Kumar Roy, Jing‐jing Wang, Yu‐ming Xu

**Affiliations:** ^1^ Department of Neurology The First Affiliated Hospital of Zhengzhou University Zhengzhou Henan China

**Keywords:** Alzheimer ’s disease, dietary intervention, exercise therapy, meta‐analysis

## Abstract

**Objective:**

The aim of this study was to investigate the clinical efficacy of exercise intervention in the treatment of patients with Alzheimer's disease (AD) by meta‐analysis.

**Methods:**

From January 2000 to January 2022, PubMed, Web of Science, Embase, CNKI, and WanFang databases were searched for all studies on the clinical efficacy of exercise intervention in the treatment of AD patients. Stata 17.0 statistical software was used for meta‐analysis.

**Results:**

Specifically, data of 983 patients were subjected to meta‐analysis, including 463 patients in the control group (conventional drug therapy) and 520 patients in the treatment group (physical exercise on the basis of conventional therapy). The results of meta‐analysis showed that Mini‐Mental State Examination (MMSE) score and Activities of Daily Living Scale (ADL) score in the treatment group were significantly higher than those in the control group. Further subgroup analysis of exercise intervention >16 weeks found that MMSE and ADL scores in the treatment group were significantly higher than those in the control group. Subgroup analysis of exercise intervention ≤16 weeks demonstrated that MMSE and ADL in the treatment group were higher than those in the control group. In addition, the treatment group had a significant lower Neuropsychiatric Inventory (NPI) score compared with the control group (SMD = –0.76, 95% CI (–1.37, –0.16), *p* = .013); subgroup analysis showed that the NPI score in the treatment group were lower than that in the control group when exercise intervention was >16 weeks [SMD = –1.01, 95% CI (–1.99, –0.04), *p* = .042] and ≤16 weeks [SMD = 0.43, 95% CI (–0.82, –0.03), *p* = .034].

**Conclusion:**

Exercise intervention can improve the neuropsychiatric symptoms, activities of daily living and cognitive function of AD patients, but the improvement is not significant in case of exercise intervention ≤16 weeks.

## Graphical abstract

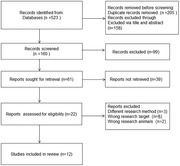

## 1 INTRODUCTION

Alzheimer's disease (AD) is a progressive neurodegenerative disease with an insidious onset. The neurons damaged first are those in parts of the brain responsible for memory, language and thinking, resulting in memory, language and thinking problems. Although these symptoms are new to the individual affected, the brain changes that cause them are thought to begin 20 years or more before symptoms start (2023 Alzheimer's disease facts and figures, 2023). Aging society has led to a rapid increase in the prevalence of AD patients (Yu et al., 2021). According to relevant institutional statistics, combined with the roughly 4.7 million Americans aged 65 and older with dementia due to Alzheimer's disease based on Alzheimer's brain changes, this would translate to approximately 10 to 12 million older Americans with Alzheimer's disease and some form of cognitive symptoms in 2023 (2023 Alzheimer's disease facts and figures, 2023). Such a big patient group means huge health care costs. Early intervention in AD patients can better control cognitive impairment and reduce functional and behavioral disorders (2023 Alzheimer's disease facts and figures, 2023).

Previous researchers have suggested higher education (Karp et al., 2004), physical exercise (Verghese et al., 2003), participation in cognitive activities (Verghese et al., 2003), and intake of antioxidant foods (Gray et al., 2003; Nicolas et al., 2001) as protective factors for AD. Results of a meta‐analysis showed that exercise intervention had a moderate positive effect on cognitive performance (effect size = 0.57) (Heyn et al., 2004). AD patients often present with emotional changes such as depression and apathy and executive dysfunction such as gait disorders in the early stages of the disease, and some researchers believe that exercise is effective in slowing gait disorders (Gras et al., 2015) and depressive mood (Arkin, 2003; Taggart, 2002). However, there is still no systematic evidence demonstrating the effectiveness of exercise intervention for AD patients. Given this lack, we collected and collated the published relevant literature and tested the efficacy of exercise for AD using a meta‐analysis.

## 2 MATERIALS AND METHODS

### 2.1 Search strategy

Published articles were searched on PubMed, Web of Science, Embase, Chinese National Knowledge Infrastructure, and WanFang databases from January 2000 to January 2022. The search keywords were as follows: “Alzheimer's Disease OR AD,” “Exercise,” and “Treatment.”

### 2.2 Inclusion criteria

(1) Study subjects: The diagnosis was in accordance with the diagnostic criteria of AD introduced in *Clinical Diagnosis and Efficacy Criteria (Chinese Edition)* (Wang, 2010). (2) Intervention measures: Patients were grouped according to different treatment methods. The control group were treated with conventional drugs, while patients in the treatment group participated in physical exercise (such as aerobic exercise, at‐home exercise, Tai Chi) on the basis of conventional treatment. (3) Study design: Randomized controlled trials (RCTs) published in relevant medical journals at home and abroad. (4) Outcome measures: At least one of the following indicators: Mini‐Mental State Examination (MMSE), Activities of Daily Living Scale (ADL), total score of Neuropsychiatric Inventory (NPI). (5) The patient's family members were informed about the study and signed the informed consent.

### 2.3 Exclusion criteria

(1) Subjects with severe critical limb ischemia, severe orthopedic diseases, severe spinal cord injury, severe muscle injury, malignant tumors, lifestyle‐limiting claudication or mental disorders caused by congenital genetic diseases. (2) Animal experiments, non‐RCTs, reviews were excluded. (3) Articles from which data required for this study could not be obtained were excluded.

### 2.4 Literature screening and data extraction

The relevant articles obtained from the search were imported into NoteExpress5.4 software, and the repeated ones were automatically eliminated by computer, and then manually eliminated by the researchers. Two researchers independently screened the literature and determined the final included literature. Then they extracted the required data by reading the titles and full text in strict accordance with the inclusion and exclusion criteria. In case of disagreement between the two researchers, a third evaluator was consulted to reach a consensus. Relevant data mainly included literature title, author, study subjects, intervention measures, and relevant scales.

### 2.5 Statistical analysis

Stata16.0 statistical software was used for meta‐analysis. Standardized mean difference (SMD) and 95% confidence interval (CI) were used to express continuous variables. Heterogeneity was tested using the χ^2^ test and *I* ^2^ statistics. If *I* ^2^ was <50% and *p* > .05, there was no statistical heterogeneity among the studies, and a fixed‐effects model was used to combine effect sizes; otherwise, a random effect model was adopted. Sensitivity analysis was performed for possible causes of heterogeneity, and funnel plot was used for publication bias analysis. Differences were considered statistically significant when *p* < .05.

## 3 RESULTS

### 3.1 Literature search results

**FIGURE 1:**
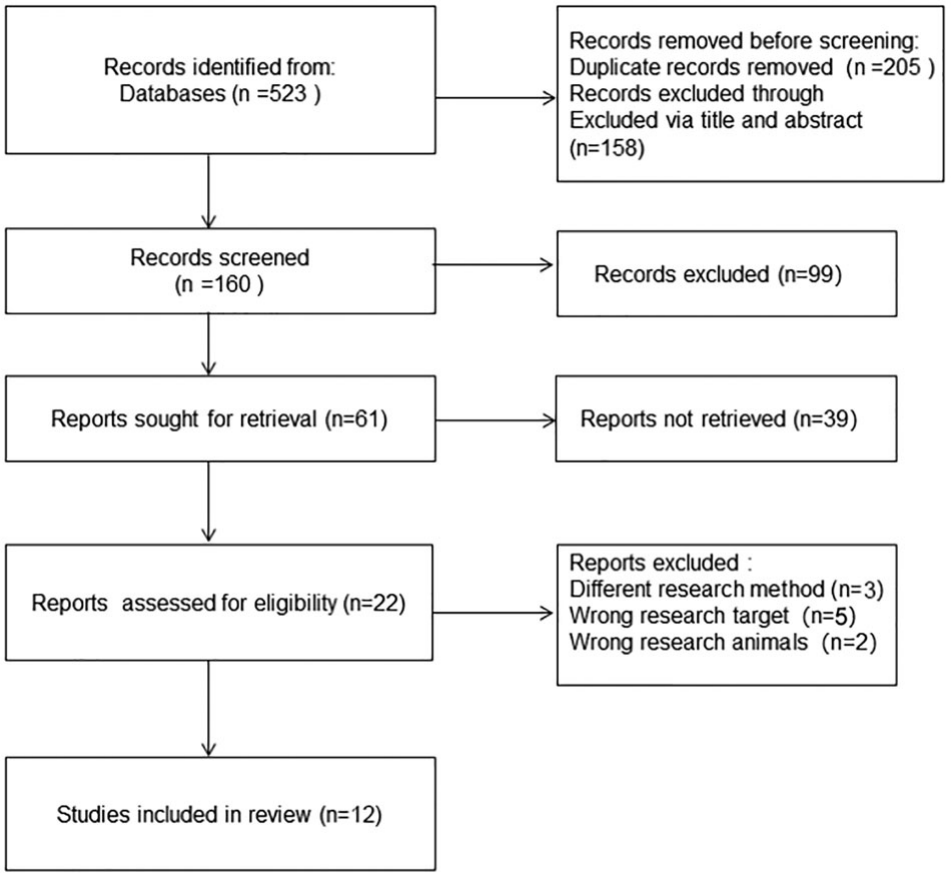
Flow chart of literature selection.

The initial search yielded 523 articles, and 205 duplicates were excluded. Then 158 articles were excluded by reading titles and abstracts, 148 were considered ineligible after reviewing the full text and data based on the inclusion and exclusion criteria. Finally, 12 studies were included (Bossers et al., 2016; Burgener et al., 2008; Dawson et al., 2019; Fleiner et al., 2017; Hoffmann et al., 2016; Holthoff et al., 2015; Huang et al., 2019; Rolland et al., 2007; Stella et al., 2011; van Santen et al., 2020; Vreugdenhil et al., 2012; Yang et al., 2022), with 983 patients (treatment group: *n* = 520; control group: *n* = 463). The literature screening process is presented in Figure 1, and the characteristics of the included studies are shown in Table 1.

**TABLE 1:** The basic characteristics of included literature

					Gender (female/male)		Age				
	Study		Country	Number (treat/cont)	Treat	Cont	Treat	Cont	Study type	Outcomes	Duration (weeks)
1	van Santen J	2020	Netherlands	73/39	36/37	16/23	79.0 ± 6.0	79.0 ± 7.0	RCT	①④⑤	24
2	Bossers WJ	2016	Netherlands	35/35	27/8	25/10	85.5 ± 5.4	85.7 ± 4.8	RCT	②	9
3	Dawson N	2019	Brazil	23/23	13/10	10/13	73.8 ± 8.5	73.9 ± 9.1	RCT	②⑤	12
4	Holthoff VA	2015	Germany	15/15	8/7	7/8	72.4 ± 4.34	70.67 ± 5.41	RCT	①②③	12
5	Fleiner T	2017	Germany	35/35	16/19	17/18	80 ± 7	80 ± 7	RCT	③	2
6	Huang N	2019	China	40/40	28/12	26/14	81.9 ± 6.0	81.9 ± 6.1	RCT	①③	40
7	Hoffmann K	2016	Denmark	107/93	51/56	36/57	69.8 ± 7.4	71.3 ± 7.3	RCT	①②③	16
8	Rolland Y	2007	French	67/67	48/19	53/14	82.8 ± 7.8	83.1 ± 7.0	RCT	②③	48
9	Stella F	2011	Brazil	16/16	/	/	/	/	RCT	③	24
10	Vreugdenhil A	2012	USA	20/20	9/11	15/5	73.5(51−83)	74.7(58−89)	RCT	①	16
14	Li Yang	2020	China	65/61	50/11	46/19	85.11 ± 6.93	85.64 ± 6.31	RCT	①②③	24
16	Sandy C	2008	USA	24/19	11/13	9/10	77.9 ± 7.9	76.0 ± 8.1	RCT	①	20

Treat: treatment group; Cont: control group; RCT: randomized controlled trial. ①: MMSE, Mini‐Mental State Examination; ②: ADL, Activities of Daily Living Scale; ③: total NPI score, Neuropsychiatric Inventory.

### 3.2 Meta‐analysis results of MMSE score

**FIGURE 2:**
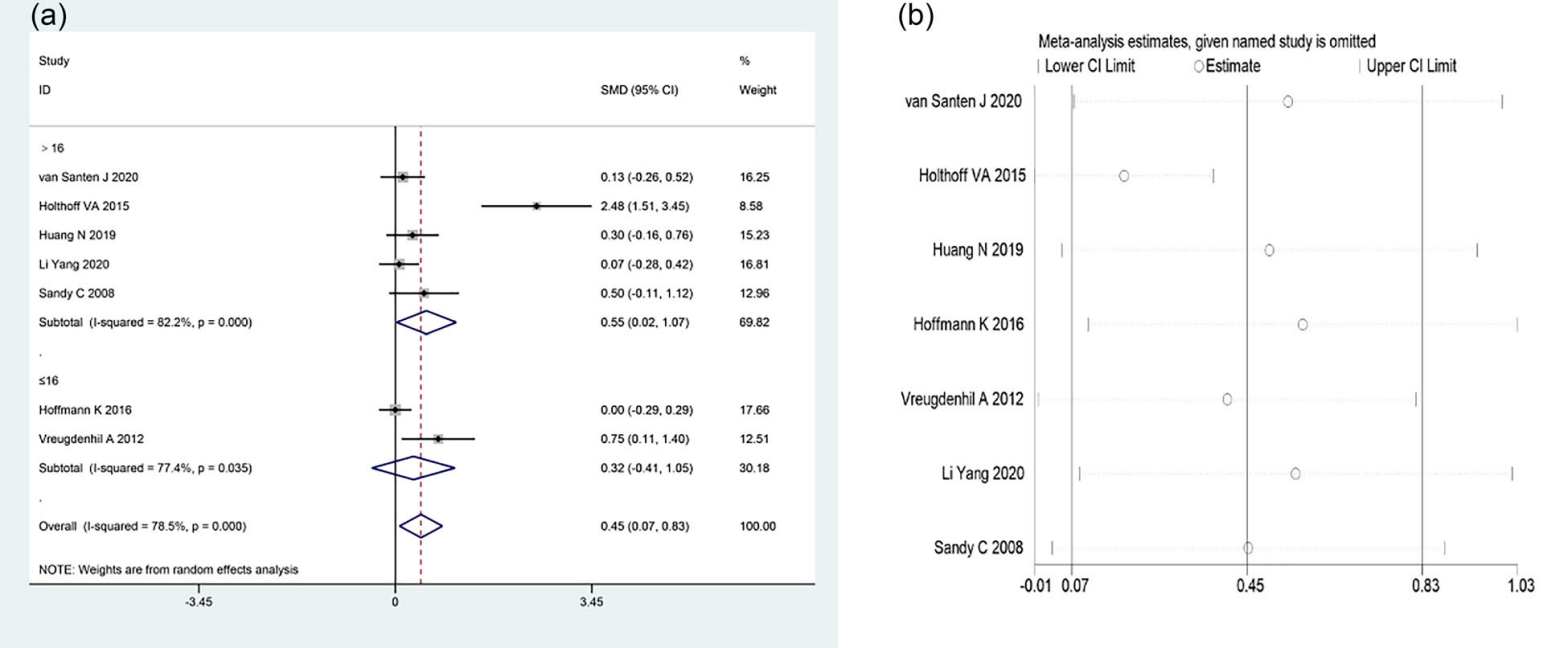
Meta‐analysis of MMSE in two groups of AD patients. (a) Forest plot comparing MMSE between the two groups. (b) Sensitivity analysis of MMSE. MMSE, Mini‐Mental State Examination.

A total of seven (Burgener et al., 2008; Hoffmann et al., 2016; Holthoff et al., 2015; Huang et al., 2019; van Santen et al., 2020; Vreugdenhil et al., 2012; Yang et al., 2022) studies compared MMSE items between the two groups in this study. Significant heterogeneity was found among studies (*I* ^2^ = 78.5%, *p* < .001), and the random‐effects model was used to combine the effect sizes. The meta‐analysis results showed that the MMSE score in the treatment group was higher than that in the control group, and the difference was statistically significant [SMD = 0.45, 95% CI (0.07, 0.83), *p* = .019; Figure 2a].

According to the experimental duration, the included studies were divided into two subgroups of ≤16 weeks and >16 weeks for further analysis. There was significant heterogeneity among studies when the exercise intervention time was >16 weeks (*I* ^2^ = 82.2%, *p* < .001), and the MMSE score in the treatment group was markedly higher than that in the control group [SMD = 0.55, 95% CI (0.02, 1.07), *p* = .041; Figure 2a]. Significant heterogeneity was also identified across studies with the exercise intervention time ≤16 weeks (*I* ^2^ = 77.4%, *p* = .035), and the treatment group showed a higher MMSE score but no marked difference compared with the control group [SMD = 0.32, 95% CI (−0.41, 1.05), *p* = .391; Figure 2a].

Sensitivity analysis was used to test the stability of the study results. The analysis results showed that the newly pooled effect size, which was obtained after removing each study one by one, was within the 95% CI of the original result, indicating that the results of this study were robust and credible (Figure 2b).

### 3.3 Meta‐analysis of ADL score

**FIGURE 3:**
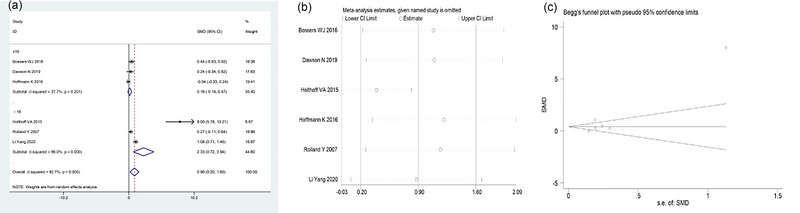
Meta‐analysis results of ADL score in two groups of AD patients. (a) Forest plot comparing ADL between the two groups. (b) Sensitivity analysis of ADL. (c) Funnel plot of ADL. ADL, Activities of Daily Living Scale.

A total of six (Bossers et al., 2016; Dawson et al., 2019; Hoffmann et al., 2016; Holthoff et al., 2015; Rolland et al., 2007; Yang et al., 2022) studies compared ADL items between the two groups in this study. The random‐effects model was adopted to combine the effect sizes because of significant heterogeneity among studies (*I* ^2^ = 92.7%, *p* < .001). The meta‐analysis results showed that the ADL score in the treatment group was significantly higher than that in the control group [SMD = 0.90, 95% CI (0.20, 1.60), *p* = .01; Figure 3a].

Further, the six studies were divided into two subgroups according to different experimental duration of each study, ≤16 weeks and >16 weeks. The results showed that there was significant heterogeneity among studies when the exercise intervention time was >16 weeks (*I* ^2^ = 96.0%, *p* < .001), and the ADL score in the treatment group was higher than that in the control group [SMD = 2.33, 95% CI (0.72, 3.94), *p* = .005; Figure 3a]. By contrast, when the exercise intervention time was ≤16 weeks, no significant heterogeneity was identified among studies (*I* ^2^ = 37.7%, *p* = .201), and the treatment group had higher ADL score, but the difference between the two groups was not statistically significant [SMD = 0.16, 95% CI (−0.16, 0.468), *p* = .326; Figure 3a].

Sensitivity analysis was carried out to test the stability of the study results. After one‐by‐one removal of the six studies, the newly pooled effect size was still within the 95% CI of the original result, indicating that the results of this study were robust and credible (Figure 3b). Begg's test was further used to detect publication bias, and no publication bias was detected (*p* = .260; Figure 3c).

### 3.4 Meta‐analysis results of NPI score

**FIGURE 4:**
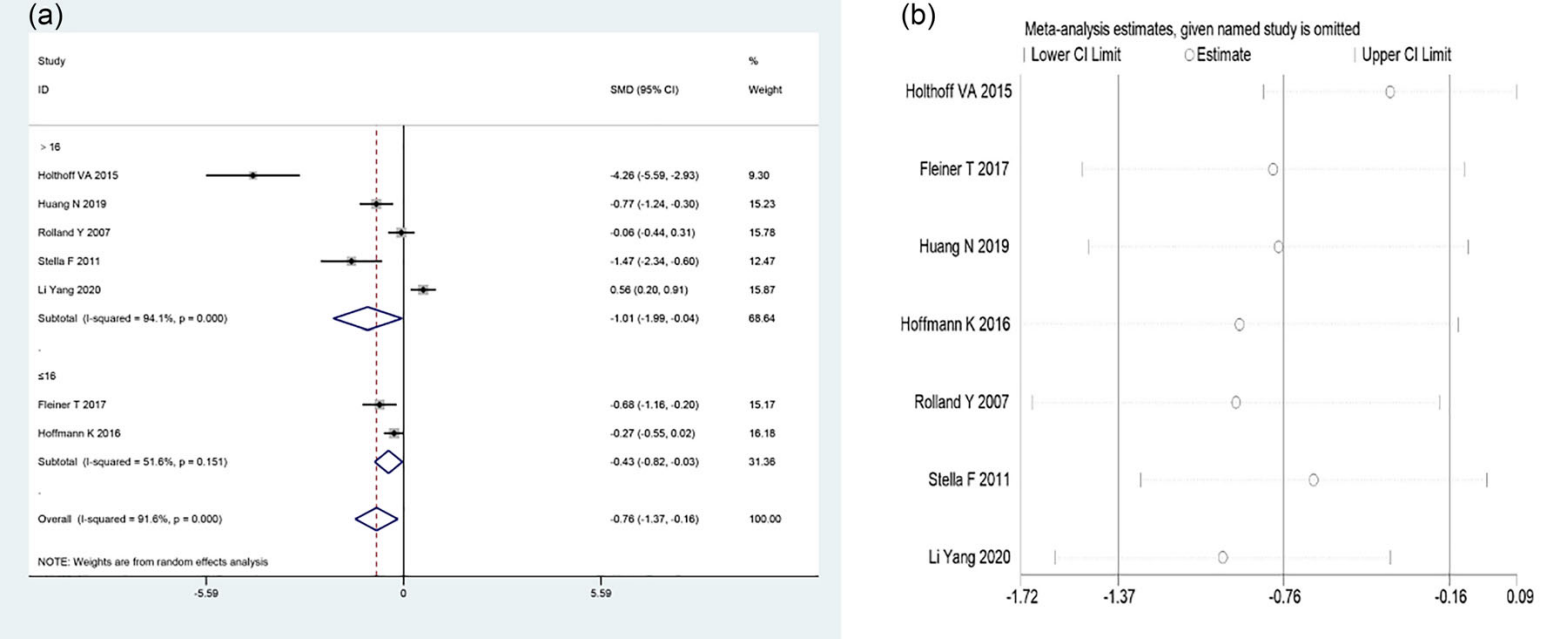
Meta‐analysis of NPI in two groups of AD patients. (a) Forest plot comparing NPI between the two groups. (b) Sensitivity analysis of NPI. NPI, Neuropsychiatric Inventory.

A total of seven (Fleiner et al., 2017; Hoffmann et al., 2016; Holthoff et al., 2015; Huang et al., 2019; Rolland et al., 2007; Stella et al., 2011; Yang et al., 2022) studies compared NPI items between the two groups in this study. The random‐effects model was employed for combining the effect sizes because there was significant heterogeneity among studies (*I* ^2^ = 91.6%, *p* < .001). The meta‐analysis results showed that the NPI score in the treatment group was significantly lower than that in the control group [SMD = −0.76, 95% CI (−1.37, −0.16), *p* = .013; Figure 4a].

In consideration of the different experimental duration of each study, ≤16 weeks and >16 weeks, the seven studies were split into two groups for further analysis. There was no significant heterogeneity among the studies when the exercise intervention time was >16 weeks (*I* ^2^ = 94.1%, *p* < .001), and the NPI score in the treatment group was significantly lower than that in the control group [SMD = −1.01, 95% CI (−1.99, −0.04), *p* = .041; Figure 4a]. When the exercise intervention time was ≤16 weeks, there was significant heterogeneity among the studies (*I* ^2^ = 51.6%, *p* = .151), and although the NPI score in the treatment group was lower than that in the control group, the difference was not statistically significant [SMD = −0.43, 95% CI (−0.82, −0.03), *p* = .034; Figure 4a].

In order to determine the stability of the study results, sensitivity analysis was carried out by one‐by‐one removal of studies, and found that the newly pooled effect size was still within the 95% CI of the original result, indicating that the results of this study were robust and credible (Figure 4b).

## 4 DISCUSSION

AD is a neurological disease commonly occurring in older adults. Reported by Alzheimer's Disease International, one more AD patient is added every 3 s, and AD treatment costs will increase to $818 billion by 2030 (Prince et al., 2015). At present, the pathogenesis of AD is not clear, and the popular hypotheses include the amyloid beta cascade hypothesis, tau hypothesis and neurovascular hypothesis (Bakota & Brandt, 2016). The amyloid beta (Aβ) cascade hypothesis suggests that an imbalance between the production and elimination of Aβ‐protein is the initial event leading to neuronal degeneration and dementia (Chong et al., 2018). The tau hypothesis is that hyperphosphorylated tau affects the stability of microtubule‐associated proteins, leading to neurofbrillary tangles and dysfunction of neurons and synapses (Chong et al., 2018). The neurovascular hypothesis considers that cerebrovascular dysfunction causes neuronal cell dysfunction and decreased Aβ‐protein clearance, resulting in cognitive impairment (Singh‐Bains et al., 2019). Attributed to the lack of clear understanding of its pathogenesis, no effective drugs were developed for the treatment of AD. Therefore, it is valuable to prevent and reduce the symptoms of AD patients through various interventions such as diet, exercise, and cognitive training. At present, many studies try to reduce the risk of AD through lifestyle intervention, reduce the symptoms of AD patients, slow the progression, and improve the cognitive function and self‐care ability of patients. Such interventions can help to ease the burden on patients' families. A prospective study of Laurin D on the risk of dementia found that people who exercised more than three times a week had half the risk of dementia compared with people who did not have exercise habits (Laurin et al., 2001). It has also been suggested that exercise prevents Aβ accumulation (Carro et al., 2006) and promotes brain‐derived neurotrophic factor expression, thereby inhibiting cognitive decline (Erickson et al., 2011). However, there is no definitive evidence to prove whether exercise intervention is effective in treating AD patients, and thus we analyzed existing studies.

We screened the relevant literature published at home and abroad in strict accordance with the inclusion and exclusion criteria, and the relevant statistics were combined for meta‐analysis. The results showed that the MMSE, ADL, and NPI scores in the treatment group were significantly better than those in the control group. Using the duration of the study as a criterion, we divided the included studies into two groups for subgroup analysis: exercise intervention time ≤16 weeks and exercise intervention time >16 weeks. The subgroup analysis showed that the treatment group was superior to the control group, but there was no statistically significant difference in MMSE score and ADL score between the two groups in the subgroup of exercise intervention ≤16 weeks. In addition, the treatment effect was better in the subgroup of exercise intervention >16 weeks, suggesting that exercise intervention can effectively improve the clinical symptoms of AD patients, but needs to be continued for at least more than 4 months. MMSE, NPI and other scales are often used to assess the neurological and psychological status of patients in neuropsychological disciplines. MMSE includes items of orientation to time and orientation to place, registration, attention and calculation, recall, language, and visual construction, which are widely used to screen and assess AD and dementia (Pinto et al., 2019). NPI, a sensitive evaluation of treatment outcome, assesses 12 common neuropsychiatric disorders in people with dementia and distress scores for caregivers (Cummings, 1997). The results of this study showed that exercise intervention was effective in improving neuropsychiatric symptoms and activities of daily living in AD patients, which is consistent with the results of a meta‐analysis of Diesfeldt and Diesfeldt‐Groenendijk (1977) and Lopez‐Ortiz et al. (2021).

This study still has some limitations. There is no sufficient literature included in this study, and particularly, there are few studies included in some evaluation indicators. Given this reason, the conclusions of this study cannot be popularized. Muticenter RCTs with large samples are required to further enhance the accuracy and reliability of the study results, and to further clarify the efficacy of exercise intervention in AD patients.

## 5 CONCLUSION

In summary, the meta‐analysis results show that exercise intervention significantly improves neuropsychiatric symptoms, psychological status, and activities of daily living in AD patients, and exercise intervention lasting more than 4 months was more effective.

## AUTHOR CONTRIBUTIONS

Sagor Kumar Roy designed the work. Jing‐jing Wang extracted and analyzed the data. Yu‐ming Xu wrote this paper. Sagor Kumar Roy, Jing‐jing Wang, and Yu‐ming Xu interpreted the results and helped to revise the manuscript. All authors read and approved the final manuscript.

## CONFLICT OF INTEREST STATEMENT

The authors declared no potential conflicts of interest with respect to the research, authorship, and/or publication of this article.

### PEER REVIEW

The peer review history for this article is available at https://publons.com/publon/10.1002/brb3.3051.

## Data Availability

All data are available upon reasonable request.
